# Supplementary material for: Hypertriglyceridemia-associated acute pancreatitis: Response to continuous insulin infusion
Source: PLoS One. 2021 Nov 30;16(11):e0260495. doi: 10.1371/journal.pone.0260495 (PMC8631651; doi:10.1371/journal.pone.0260495)
Supplement: Attachment — Submitted filename: PLOS One Reviewer Responses 10-22-21.docx [file pone.0260495.s002.docx]

Journal Requirements:

1. Please ensure that your manuscript meets PLOS ONE's style requirements, including those for file naming. The PLOS ONE style templates can be found at 
https://journals.plos.org/plosone/s/file?id=wjVg/PLOSOne_formatting_sample_main_body.pdf and 
<https://journals.plos.org/plosone/s/file?id=ba62/PLOSOne_formatting_sample_title_authors_affiliations.pdf>

***Data included as Supplemental Materials***

2. Please provide additional details regarding participant consent. In the ethics statement in the Methods and online submission information, please ensure that you have specified (1) whether consent was informed and (2) what type you obtained (for instance, written or verbal, and if verbal, how it was documented and witnessed). If your study included minors, state whether you obtained consent from parents or guardians. If the need for consent was waived by the ethics committee, please include this information.

If you are reporting a retrospective study of medical records or archived samples, please ensure that you have discussed whether all data were fully anonymized before you accessed them and/or whether the IRB or ethics committee waived the requirement for informed consent. If patients provided informed written consent to have data from their medical records used in research, please include this information.

***Information about de-identified data added to Methods section, page 4.***

3. We suggest you thoroughly copyedit your manuscript for language usage, spelling, and grammar. If you do not know anyone who can help you do this, you may wish to consider employing a professional scientific editing service.

Whilst you may use any professional scientific editing service of your choice, PLOS has partnered with both American Journal Experts (AJE) and Editage to provide discounted services to PLOS authors. Both organizations have experience helping authors meet PLOS guidelines and can provide language editing, translation, manuscript formatting, and figure formatting to ensure your manuscript meets our submission guidelines. To take advantage of our partnership with AJE, visit the AJE website (http://learn.aje.com/plos/) for a 15% discount off AJE services. To take advantage of our partnership with Editage, visit the Editage website (www.editage.com) and enter referral code PLOSEDIT for a 15% discount off Editage services.  If the PLOS editorial team finds any language issues in text that either AJE or Editage has edited, the service provider will re-edit the text for free.

Upon resubmission, please provide the following: 
● The name of the colleague or the details of the professional service that edited your manuscript
● A copy of your manuscript showing your changes by either highlighting them or using track changes (uploaded as a *supporting information* file)
● A clean copy of the edited manuscript (uploaded as the new *manuscript* file)

[Note: HTML markup is below. Please do not edit.]

Reviewers' comments:

Reviewer's Responses to Questions

**Comments to the Author**

1. Is the manuscript technically sound, and do the data support the conclusions?

The manuscript must describe a technically sound piece of scientific research with data that supports the conclusions. Experiments must have been conducted rigorously, with appropriate controls, replication, and sample sizes. The conclusions must be drawn appropriately based on the data presented.

Reviewer #1: Yes

Reviewer #2: Yes

Reviewer #3: Yes

2. Has the statistical analysis been performed appropriately and rigorously?

Reviewer #1: Yes

Reviewer #2: Yes

Reviewer #3: Yes

3. Have the authors made all data underlying the findings in their manuscript fully available?

The [PLOS Data policy](https://nam12.safelinks.protection.outlook.com/?url=http%3A%2F%2Fwww.plosone.org%2Fstatic%2Fpolicies.action%23sharing&data=04%7C01%7Cbtahsin%40cookcountyhhs.org%7C25216a2062b54df2308f08d977972912%7C3b922295e886417faaa84e4c4f069d82%7C0%7C0%7C637672313396898939%7CUnknown%7CTWFpbGZsb3d8eyJWIjoiMC4wLjAwMDAiLCJQIjoiV2luMzIiLCJBTiI6Ik1haWwiLCJXVCI6Mn0%3D%7C1000&sdata=oLsNtB2NpWobu%2FXXslVSWMx1M1vzdkJ1UJmaivMX9Cw%3D&reserved=0) requires authors to make all data underlying the findings described in their manuscript fully available without restriction, with rare exception (please refer to the Data Availability Statement in the manuscript PDF file). The data should be provided as part of the manuscript or its supporting information, or deposited to a public repository. For example, in addition to summary statistics, the data points behind means, medians and variance measures should be available. If there are restrictions on publicly sharing data—e.g. participant privacy or use of data from a third party—those must be specified.

Reviewer #1: No

Reviewer #2: Yes

Reviewer #3: Yes

4. Is the manuscript presented in an intelligible fashion and written in standard English?

PLOS ONE does not copyedit accepted manuscripts, so the language in submitted articles must be clear, correct, and unambiguous. Any typographical or grammatical errors should be corrected at revision, so please note any specific errors here.

Reviewer #1: Yes

Reviewer #2: Yes

Reviewer #3: Yes

5. Review Comments to the Author

Please use the space provided to explain your answers to the questions above. You may also include additional comments for the author, including concerns about dual publication, research ethics, or publication ethics. (Please upload your review as an attachment if it exceeds 20,000 characters)

Reviewer #1: The aim of this retrospective case series is to analyze the outcome of to continuous insulin infusion in hypertriglyceridemia-associated acute pancreatitis. It was concluded that continuous insulin infusion was effective in reducing serum triglyceride level in the majority of patients. Presence of diabetes, higher BMI and initial triglyceride slowed the time needed to effectively reduce serum triglyceride level.

There are many case reports and series demonstrating the effectiveness of insulin in lowering triglyceride level. This study provides data about the quantity of insulin and the duration required to reduce effectively the serum triglyceride level.

Criticisms:

1. Fasting and intravenous hydration applied in the treatment of acute pancreatitis rapidly and greatly reduces serum triglyceride level and can be as effective as intravenous insulin. Provide data about the applied intravenous fluid therapy in the first days of your patients. Intravenous hydration can be a confounding factor in this study. Please, discuss it.

***In our ICUs, such patients would receive IV fluids (******either 0.9% or 0.45% saline) based on hydration parameters. We did not include these data since the IV fluid therapy is consistently implemented with hydration information added in the Methods section, page 4.***

2. Was dextrose infusion applied as part of the parenteral fluid therapy in the study? Please, provide details in the Methods.

***There were no indications in the blood glucose data that any patients would have required dextrose infusions, therefore not mentioned in the Method section.***

3. Could you give data about the severity and outcome of acute pancreatitis especially in the RP and NR, SRP and RRP group, respectively?

***This study was focused on the impact of CII on HTG reduction. Data to assess severity of AP were not collected. However, based on admission and discharge dates, all patients survived and were discharged home. Sentence added in Results, page 10.***

4. What is the 75th percentile in this data from table 2? 4 [0.5]

***Fixed typo – 4 [0, 5]***

5. Page 12: ‘Figure 1: Consort Table’. Some contradiction: is it a figure or table? ‘Patient’s flow chart’ is suggested to be used.

***Figure 1 title changed to Consort Chart***

6. Page 22: ‘Figure 1’ is supposed to be Figure 2. What is the meaning of gosl? Please use the word time/Time, insulin/Insulin consistently.

***Figure 1 changed to Figure 2 and typos/inconsistencies fixed.***

7. The style of references does not follow the requirements of the journal.

***Reference style updated.***

8. There are many typos in the manuscript. Some of them:
Abstract: kg/m2…instead of …kg/m 2…
Abstract: …HbA1c… instead of …HbA1C…
Page 10: …abdominal pain… instead of…. pain abdomen…
Repeatedly:…. p = 0.130… instead of …p 0.130…
Table 2 title: …. less than 36 hours vs 36 hours …instead of … less than vs 36 hours …
Table 2:… 36 hours or more… instead of … 36 hours or More …
Table 2: … (hours) … instead of … hours)…

***Typos corrected.***

Reviewer #2: 1. Please review abstract. Currently not able to be interpreted easily.

***Abstract revised***.

2. "CII is an effective modality of therapy to reduce acutely the TG levels" - please review syntax

***Sentence on page 3 revised to: CII is an effective modality of therapy to*** ***reduce TG levels rapidly.***

3. "AP was defined per Atlanta classification of acute
pancreatitis 2012 as any two of the three following criteria: pain abdomen (acute onset, persistent, severe, epigastric pain often radiating to the back), pancreatic enzymes (serum lipase or amylase) level at least 3 times greater than the upper limit of normal, computed tomography (CT) or magnetic resonance imaging (MRI) evidence of acute pancreatitis" - please correct to abdominal pain

***Corrected.***

4. Why were pregnant women excluded from this study?

***Pregnant women were excluded to eliminate pregnancy as a confounding variable. At the same time, no patients with pregnancy were in the initial study group prior to exclusion. In this study of acute pancreatitis, no pregnant patients with acute pancreatitis presented.***

5. "Secondary outcomes were time durations to achieve TG-50% (half of the respective initial TG) and time durations to achieve TG-goal" - please take out durations

***Corrected.***

6. What is your institution's protocol about rate and concentration of dextrose delivery? This will affect insulin doses

***No information about rate and concentration of dextrose delivery is needed so there were no indications in the blood glucose data that any patients would have required dextrose infusions.***

7. "The four patients who did not reach TG-goal (NR, non-responders) had a long time to initiation of CII (3 [0, 5.5] vs 10 [2.2, 37.2] hr, p 0.130) but otherwise did not differ from RP" - p value suggests this is not significant

***As suggested, this statement was deleted.***

Reviewer #3: In this manuscript authors investigated efficacy of continuous insulin insfusion on lowering triglyceride levels in patients with hypertriglyceridemic pancreatitis. They also search for the factors that can predict treatment success. This study gives a new insight how insulin, as a triglyceride lowering agent, could be used in patients with hypertriglyceridemic pancreatitis and which parameters could help in titration of therapy. In the current literature this kind of data are lacking so this manuscript is of great importance from the clinical and research points of view.
Introduction provides good and concise background of the topic. The objective is clearly defined.
Methodology of the study is well designed but continuous insulin infusion protocol could be explained in a more detailed way (quantity of insulin in infusion, type and volume of solution, frequency of glucose checks, goal glucose levels and adjustment of infusion rate).

***More details were added to the Methods section, page 4.***

Other components of methodology, inclusion/exclusion criteria and outcomes are thoroughly written.
Results are well presented. Tables and figures are clear, informative and easy to follow. The data provide enough evidence to make a conclusions.
Discussion gives good perspective of current knowledge and how findings of this study can upgrade clinical practice. Conclusions are well presented and supported by the data.
The literature cited is relevant to the study.

6. PLOS authors have the option to publish the peer review history of their article ([what does this mean?](https://nam12.safelinks.protection.outlook.com/?url=https%3A%2F%2Fjournals.plos.org%2Fplosone%2Fs%2Feditorial-and-peer-review-process%23loc-peer-review-history&data=04%7C01%7Cbtahsin%40cookcountyhhs.org%7C25216a2062b54df2308f08d977972912%7C3b922295e886417faaa84e4c4f069d82%7C0%7C0%7C637672313396898939%7CUnknown%7CTWFpbGZsb3d8eyJWIjoiMC4wLjAwMDAiLCJQIjoiV2luMzIiLCJBTiI6Ik1haWwiLCJXVCI6Mn0%3D%7C1000&sdata=My4Geoqli0onlCHklbv5xdVqeYy7pXUkLh%2FuRO5R%2BGA%3D&reserved=0)). If published, this will include your full peer review and any attached files.


If you choose “no”, your identity will remain anonymous but your review may still be made public.


**Do you want your identity to be public for this peer review?** For information about this choice, including consent withdrawal, please see our [Privacy Policy](https://nam12.safelinks.protection.outlook.com/?url=https%3A%2F%2Fwww.plos.org%2Fprivacy-policy&data=04%7C01%7Cbtahsin%40cookcountyhhs.org%7C25216a2062b54df2308f08d977972912%7C3b922295e886417faaa84e4c4f069d82%7C0%7C0%7C637672313396908894%7CUnknown%7CTWFpbGZsb3d8eyJWIjoiMC4wLjAwMDAiLCJQIjoiV2luMzIiLCJBTiI6Ik1haWwiLCJXVCI6Mn0%3D%7C1000&sdata=A2Grb%2B7b%2B7xRhPT76EENndBhk3Ht1DDg%2FaifqZKFhTw%3D&reserved=0).

Reviewer #1: No

Reviewer #2: No

Reviewer #3: No
